# Cross-talk between the inflammatory response, sympathetic activation and pulmonary infection in the ischemic stroke

**DOI:** 10.1186/s12974-014-0213-4

**Published:** 2014-12-24

**Authors:** Pawel J Winklewski, Marek Radkowski, Urszula Demkow

**Affiliations:** Institute of Human Physiology, Medical University of Gdansk, Tuwima Street 15, 80-210 Gdansk, Poland; Department of Immunopathology of Infectious and Parasitic Diseases, Medical University of Warsaw, Warsaw, Poland; Department of Laboratory Diagnostics and Clinical Immunology of Developmental Age, Medical University of Warsaw, Warsaw, Poland

**Keywords:** Stroke, Brain injury, Immune response, Sympathetic nervous system, Lung injury, Pneumonia

## Abstract

The immune system response and inflammation play a key role in brain injury during and after a stroke. The acute immune response is responsible for secondary brain tissue damage immediately after the stroke, followed by immunosuppression due to sympathetic nervous system activation. The latter increases risk of infection complications, such as pneumonia. The pneumonia-related inflammatory state can release a bystander autoimmune response against central nervous system antigens, thereby initiating a vicious circle. The aim of this review is to summarize the relationship between ischemic stroke, sympathetic nervous system activation and pulmonary infection.

## Introduction

It is estimated that pulmonary and urinary tract infections occur in up to one third of patients suffering from ischemic stroke within the first days after the vascular event [1,2]; however, frequency of these infections varies between studies [3]. The largest trial undertaken to investigate the effect of pneumonia on mortality in hospitalized patients with acute stroke reported a 27% 30-day mortality rate in 635 patients due to pneumonia compared to 4% death rate among patients free from severe respiratory infections (*P* < 0.001) [4]. The most important predictors of post-stroke infection include older age, stroke severity (that is National Institutes of Health Stroke Scale of more than 16 in 0 to 42 scale, Barthel Index of less than 5 in 0 to 20 scale, Glasgow Coma Scale score of less than 9), larger area of the infarct, and dysphagia [4,5].

Dysphagia is a well-recognized risk factor for post-stroke pneumonia. Traditionally, it has been explained by abnormal dopamine transmission and/or a decrease of substance P in the gastrointestinal tract resulting in the impairment of the swallowing reflex [6]. Dysphagia is a frequent indication for tube feeding as a prophylactic procedure against post-stroke pneumonia, however, with very limited effects [7]. As none of the modalities used to treat dysphagia provide efficient protection against post-stroke pneumonia, it is warranted to discern alternative etiologies for the respiratory insult, including a stroke-induced systemic immune response [3]. This assumption corresponds with the observation that up to half of the patients presenting with pneumonia do not aspirate [8]. Furthermore, Prass *et al*. [9] demonstrated that experimental stroke can promote bacterial aspiration from harmless intranasal colonization, leading to pneumonia. Prevention of infections by β-adrenergic receptors (β-ARs) blockade suggests that immunodepression due to sympathetic nervous system (SNS) hyperactivity is essential for the development of aspiration-induced pneumonia [10]. The insular cortex strokes associated with excessive sympathetic stimulation increase the risk of post-stroke pneumonia in humans [11].

Acute immune activation after the stroke is responsible for secondary brain injury [12,13]. It is followed by immunosuppression, consequently increasing the risk of infections such as pneumonia. Recently, multiple experimental and clinical studies were performed to explain the relationship between infection after the stroke and immunosuppression induced by the SNS [9,10,14].

Ischemic stroke gives rise to an intense activation of the SNS and the release of catecholamines [9,10]. Primary (bone marrow and thymus) and secondary (spleen and lymph nodes) lymphoid organs are abundantly innervated by autonomic, mostly sympathetic efferent fibers. The SNS primary neurotransmitter - norepinephrine is released into the lymphoid tissue and modulates the function of immune cells [15]. The stroke-induced SNS activation is responsible for lymphopenia, impaired function of monocytes, shift from Th1 to Th2 cytokine production and increased lymphocyte apoptosis, observed in the affected patients [9-11]. Pharmacological inhibition of the peripheral SNS activation with a beta-blocker was shown to prevent immunosuppression in experimental stroke [10]. A retrospective analysis showed that the use of beta-blockers is associated with reduced risk of early death in patients with ischemic stroke [16]. The aim of this review is to discuss the relationship between ischemic stroke, SNS activation and pulmonary infection.

### Immune system in stroke

Ischemic cerebral stroke results from a transient or permanent reduction in local blood flow. Focal cerebral ischemia initiates a complex process including the release of neurotransmitters and activation of the immune system. Recently, the inflammatory mechanisms involved in the pathogenesis of brain injury and brain repair mechanisms (that is neural plasticity) have received considerable attention [12,13].

The response of the immune system to stroke is biphasic, with early transient activation lasting up to 24 hours [17,18] followed by systemic immunodepression, called brain-induced immunosuppression [14,18]. The suppression of cell-mediated immunity (lymphopenia, monocyte deactivation, shift from Th1 to Th2 cytokine production) 72 hours after experimental stroke is often associated with spontaneous bacteremia and pneumonia [10]. Prass *et al*. [10] demonstrated that reduced IFN-γ production as well as impaired natural killer (NK) and T cell response are the critical stroke-related failures in antibacterial defense. Other animal studies demonstrated decrease in IL-10 release by endotoxin-stimulated whole blood cells after infusion of IL-1β into the brain. This effect was attributed to activation of the hypothalamic-pituitary-adrenal (HPA) axis and the sympatho-adrenal medullary axis [19].

### Spleen

Offner *et al*. [17] observed that 6 and 22 hours after experimental stroke in mice, activated spleen cells secreted significantly higher amounts of TNF-α, IFN-γ, IL-6, monocyte chemotactic protein 1 (MCP-1), and IL-2 than splenocytes in control mice. Moreover, stimulated splenocytes from stroke-injured animals strongly expressed chemokines and chemokine receptors (CCR), including macrophage inflammatory protein 2 (MIP-2) and CCR2, CCR7 and CCR8 6 hours after stimulation; MIP-2, IFN-γ-induced protein 10 (IP-10), CCR1, and CCR2 were expressed 22 hours after stimulation. Experimental stroke increased the release of inflammatory cytokines from activated T cells [17]. Twenty-four hours later, a higher CD4/CD8 T cell ratio and enhanced production of TNF-α and IFN-γ by blood and spleen lymphocytes were reported [18].

These observations correspond with the finding of Hardy *et al*. that phenylephrine (an α_1−_AR agonist) induces contractions of the spleen in a dose-dependent manner [20]. Therefore, the spleen may serve as a dynamic reservoir of leukocytes, which are mobilized by catecholamines and migrate to the injured brain. Interestingly, the reduction in spleen size could be inhibited by α_1_-AR antagonist prazosin. However, this effect did not influence the infarct size, demonstrating that these two events are not directly linked to each other [21]. Offner *et al*. [22] suggested that spleen shrinkage was due to profound apoptosis of the lymphocyte population. However, it seems unlikely that even an extensive apoptosis would eliminate lymphocytes massively to reduce the spleen size by 40%, yet preserve their relative count in the organ [22]. Moreover, according to another report, the spleen may release large numbers of platelets upon low-dose epinephrine infusion [23]. The platelets may, as described below, significantly contribute to the development of an acute inflammatory response in the brain.

### Liver

Wong *et al*. [24] reported that noradrenergic innervation of the liver modulates the function of intrahepatic invariant NK T cells accounting for systemic immune suppression after experimental ischemic stroke. Invariant NK T cells are a unique subset of lymphocytes that express a repertoire of highly restricted T cell receptors. These receptors recognize microbe-specific or endogenous glycolipids presented by the major histocompatibility complex class I-like molecule CD1d. They are believed to play a critical role in host defense to various microbial pathogens as they are capable for a rapid release of cytokines (such as INF- γ) and chemokines. These mediators contribute considerably to the recruitment and activation of various leukocyte subsets, including neutrophils and macrophages. Invariant NK T cells reside in the vasculature, mainly in the liver and spleen [25]. Either blockade of β-ARs with propranolol or depletion of noradrenergic nerve terminals in the liver boost the immune response by increasing IFN-γ secretion from invariant NK T cells. Conversely, injection of noradrenaline into the liver downregulates inflammatory mechanisms by attenuating the functions of invariant NK T cells [24].

### Cerebral vessels

Cerebral ischemia induces the expression of TNF-α, IL-1β, IL-6, inducible nitric oxide synthase (iNOS) and TNF-α receptors in the endothelium of cerebral arteries [26,27]. Furthermore, TNF-α, IL-1β, epidermal growth factor (EGF), and basic fibroblast growth factor (bFGF) can modify the expression and function of endothelin receptors in cultured rat middle cerebral arteries [28]. Upregulation of the endothelin receptor in experimental stroke may be due to the effect of cytokines and growth factors [27]. This mechanism may facilitate cerebral vessel contraction *in vivo*, and consequently worsen cerebrovascular risk by lowering cerebrovascular reserves and increase the vulnerability of the brain to cerebral ischemia [29].

Despite intensive studies, the physiological role of the SNS in the regulation of cerebral blood flow remains far from clear [30-32]. Studies demonstrating SNS involvement in the regulation of cerebral blood flow [33,34], indicate that the stroke-induced SNS activity fluctuations are a significant factor of impaired cerebral autoregulation [35,36]. In particular, massive release of catecholamines may further amplify the effects of upregulated endothelin receptor. Thus, the pro-inflammatory signals and SNS activation promote microvascular occlusion and may increase the infarct size [37]. Regulation of cerebral blood flow might also be impaired by the administration of tissue plasminogen activator used to improve the perfusion of ischemic areas [38].

Early restoration of blood flow remains the treatment of choice for limiting brain injury following ischemic stroke. While reperfusion of the ischemic brain is desirable, tissue damage may result from reperfusion *per se*. The accumulation of T cells in post-capillary venules is accompanied by the recruitment of adherent platelets [39]. As mentioned above, large numbers of platelets are released from the spleen following sympathetic stimulation [23]. A novel concept of ‘thrombo-inflammation’ suggests that there is a strong correlation between thrombus formation and inflammation, and early platelet adhesion/activation may exacerbate infarct development following cerebral ischemia and reperfusion [40,41].

### Blood–brain barrier breakdown

**Figure 1:**
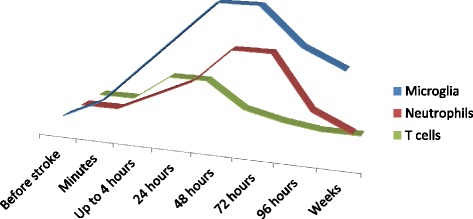
**A schematic time-course of the microglial cells, neutrophils and T lymphocytes after ischemic stroke.** Percent increase in cell count after experimental stroke in ischemic hemisphere has been estimated from the scientific literature by the authors [10,47-50,61].

A schematic time-course of the microglial cells, neutrophils and T lymphocytes after stroke was shown in Figure 1.

Microglial cells, the resident macrophages of the brain, are activated within minutes of the onset of ischemia. They produce a plethora of pro-inflammatory mediators, including reactive oxygen species (ROS), IL-1β, IL-6, and TNF-α [42,43], which further exacerbate tissue damage [44]. Microglial cell activation plays a crucial role in the increase of blood–brain barrier permeability and the early infiltration of circulating leukocytes into the brain [45,46]. The post-ischemic peak of microglial proliferation is observed at 48 to 72 hours after focal cerebral ischemia and may last for several weeks after the initial injury [47,48].

In contrast to rapid response of resident microglia, blood-derived leukocytes are recruited to the brain tissue usually with a delay of hours to a few days. Neutrophils are among the first leukocytes to infiltrate the ischemic brain (30 minutes to a few hours of focal cerebral ischemia), peak (24 to 72 hours), and then decrease rapidly [49,50]. On the other hand, Gelderblom *et al*. [51] questioned this scenario, suggesting that the infiltration of other inflammatory cells, including macrophages, lymphocytes, and dendritic cells may precede neutrophil influx. Activation of spleen neutrophils under physiological conditions is modulated by the SNS [52]. Spleen contraction induced by the SNS activation and blood–brain barrier breakdown after ischemic stroke may facilitate their migration to the injured brain. Nevertheless, the clear role of neutrophils in the pathogenesis of ischemic stroke is far from being elucidated.

T lymphocytes are central to the development of a sustained inflammatory response and accumulate in the ischemic brain within a few hours of reperfusion [53,54]. T cells may contribute to brain injury through several potential mechanisms. Cytokines and chemokines released by T helper (T_H_) and T cytotoxic (T_C_) cells (which are IL-12, IL-17, and IL-23) may exacerbate post-stroke inflammation and increase infarct area [55,56]. Furthermore, cytokines and chemokines are likely to increase the expression of vascular adhesion molecules and attract other immune cells into the brain resulting in widespread apoptosis [57]. CD3+ cells are a major source of INF-γ and perforin in the ischemic hemisphere [58] whereas T_C_ cells directly induce cell necrosis and apoptosis by the release of cytotoxins or activation of the Fas receptor [59]. Finally, T lymphocytes may release large amounts of ROS [53]. Thus, it is commonly accepted that T cells are a major source of pro-inflammatory cytokines in the brain after the stroke [60].

In patients with the ischemic event, the number of T, B and NK lymphocytes in the circulation is quickly reduced [10,61], possibly as an endogenous protective mechanism attenuating local inflammatory process in the brain [61]. A profound systemic immunodepression occurs as soon as 12 hours after the stroke and persists for several weeks [10,62]. This immunodepression is due to SNS and HPA axis hyperactivity [10] and is mediated by β2 adrenergic receptors [63]. According to a recent emerging concept, the immunodepression after stroke is attributable to the increase in the number of systemic CD4^+^CD25^+^FoxP3^+^ regulatory T (Treg) cells [18,22]. Increased percentage of Treg cells in bone marrow and their mobilization to the circulation is facilitated by the stroke-induced activation of the SNS via β2- and β3-ARs signaling, respectively [64]. Systemic immunodepression is not unique to ischemic stroke, and can also follow another brain injury including traumatic events or brain surgery [14]. Treg-mediated immunosuppression and increased susceptibility to subsequent bacterial infections can be attributed to the deficit of IFN-γ resulting in insufficient activation of phagocytic cells at the site of infection [64].

### Brain-lung cross-talk

Neurologic conditions that cause abrupt, rapid and extreme elevation in intracranial pressure give rise to an intense activation of the SNS and the release of catecholamines [65]. The non-neurological consequences of brain injury show similarities regardless of the brain insult and, in particular, include neurogenic pulmonary edema (NPE) [66].

### Acute lung injury (ALI) as a consequence of acute brain injury

ALI is a diffuse heterogeneous lung injury characterized by hypoxemia, non-cardiogenic pulmonary edema, low lung compliance and widespread capillary leakage [67]. It has been demonstrated that approximately one third of patients with acute brain injury develop ALI. Patients with concomitant ALI seem to have a worse prognosis [68,69]. The pathogenesis of ALI includes NPE, the activation of neutrophils, inflammatory mediator release, alveolar/capillary barrier damage, activation of the coagulation system, surfactant depletion and infections [64]. Brain injury might also increase lung vulnerability to subsequent deleterious mechanical or ischemia-reperfusion injury, thereby increasing the risk of subsequent respiratory failure [70].

NPE is a well-recognized complication of central nervous system (CNS) injury [66]. The proposed mechanisms governing NPE include a massive sympathetic discharge following severe brain injury causing alveolar/capillary barrier damage, intra-alveolar accumulation of protein-rich edema fluid, hemorrhage, and atelectasis. The precise origin of sympathetic outflow has not yet been identified. Nevertheless, it is believed that the ‘NPE trigger zones’ include the hypothalamus and the medulla, specifically areas A1 and A5, the nuclei of the solitary tract, and the area postrema [71]. It has been hypothesized that a massive SNS discharge following CNS injury directly affects the pulmonary vascular bed via α- and β-ARs, leading to isolated pulmonary venoconstriction and/or endothelial damage. This theory, termed ‘pulmonary venule adrenergic hypersensitivity’, can explain the direct impact of nervous system on the pulmonary endothelium, not intermediated by hemodynamic changes [66]. Nevertheless, the most established is the ‘blast theory’, which combines hemodynamic and high permeability mechanisms [72]. According to Rassler *et al*. the severity of pulmonary edema evoked by experimental intravenous injection of catecholamines correlates with pulmonary venous hypertension [73]. Moreover, Theodore and Robin (1975) proved that an increase in the pulmonary capillary wedge pressure augmented fluid filtration into the pulmonary interstitium [72].

There is mounting evidence that a systemic inflammatory response may also play a pivotal role in the development of pulmonary dysfunction after traumatic brain injury [74,75] or subarachnoid hemorrhage [76]. It seems that the initial sympathetic stimulation originating from the SNS may trigger a cascade of events in the lungs, including endothelial cell dysfunction and a systemic inflammatory response with pulmonary infiltration of neutrophils and cytokine release [75]. Cerebral hemorrhage increases the expression of intracellular adhesion molecules in both brain and lungs, resulting in progressive neutrophil recruitment to the lung interstitium and alveolar spaces with the disruption of alveolar structures [77]. It has been proposed that sympathetic activation of the lungs may provoke local release of cytokines and chemokines [76]. Furthermore, in experimental settings, catecholamines seem to activate NFκB in macrophages with subsequent inflammatory cytokine production (IL-6, TNF-α and IL-1β) in the lungs in a dose-dependent manner. Upregulation of the phagocyte response via α2-ARs enhances the acute inflammatory response [78].

In rats, NPE induced by lethal injury of the brain is characterized by increase of pro-inflammatory cytokines, in particular IL-6, and accumulation of immune cells in bronchoalveolar lavage fluid [79,80]. The histological assessment of lung tissue in this model reveals infiltration of inflammatory cells like neutrophils within interstitial spaces [80]. Experimental studies showed that norepinephrine infusion enhances expression of pro-inflammatory cytokines such as IL-6, IL-1α and IL-1β in lung tissue and in bronchoalveolar lavage fluid [73]. The systemic increase in catecholamines augments the release of IL-6 from lung macrophages via β_2_-ARs, which in turn contributes to the development of a hypercoagulable state [81]. Avlonitis *et al*. [79] demonstrated that administration of α-adrenergic antagonists prevented the development of inflammatory lung injury by reducing systemic inflammation and preserving capillary-alveolar membrane integrity. Thus, the experimental data strongly supports the hypothesis that the systemic increase of catecholamines due to the SNS activation contributes to local inflammation in lungs and impairs capillary-alveolar membrane integrity via α- and β-ARs located on phagocytes and in the pulmonary vascular bed.

### Stroke and lung injury

Mascia proposed a ‘double hit model’ to explain the pathogenesis of lung failure associated with acute brain injury [75]. The model was originally developed for traumatic brain injury; however, it shares several typical features with stroke-induced respiratory distress syndrome. Although, stroke pathophysiology significantly differs from traumatic injury brain insult it is a starting point for the local inflammation. It was shown that severe ischemic stroke was associated with an increase in the total lung water content, which might have been of neurogenic origin [82]. Nevertheless, even if the fully developed NPE does not emerge after ischemic stroke, an increase in the permeability of the alveolar/capillary barrier and local inflammatory state may favor subsequent infections.

Modulation of bulbar reflexes, drowsiness, bed rest and decreased ventilation rate combined with deep immunosuppression after stroke may trigger the development of pneumonia. Clinical data indicate that *Streptococcus pneumoniae*, the most common cause of bacterial community-acquired pneumonia [83] is also a predominant agent responsible for respiratory infections, prolonged hospitalization, and lethality in stroke patients [3]. By employing animal studies it was shown that nasal inoculation of as little as 200 colony-forming units of *Streptococcus pneumonia* causes severe pneumonia and bacteremia after experimental stroke, whereas at least 200,000 colony-forming units are needed to induce pneumonia in controls [9]. Other common pathogens such as *Chlamydophila pneumoniae* and *Haemophilus influenzae* also contribute to the development of severe infectious complications [83].

### Post-stroke autoimmune responses

The teleological explanation for post-stroke immunosuppression is that it prevents the CNS from developing adaptive immune responses directed against self-components. After stroke, due to brain–blood barrier breakdown, lymphocytes infiltrate the ischemic brain allowing for contact with CNS antigens from various CNS cell types like neurons, astrocytes and oligodendrocytes. Furthermore, the increase of antigens expression such as myelin basic protein (MBP), neuron specific enolase, S-100 or glial fibrillary acidic protein (GFAP) are observed. These components become ‘visible’ to the peripheral immune system, and indeed antigen presentation within days of stroke onset has been reported in cervical lymph nodes [84].

In experimental studies of severe stroke, not associated with infection, Th1 responses to MBP are uncommon. Nevertheless, the tendency to develop a Th1 response to MBP could be increased by lipopolysaccharide (LPS)-mediated induction of a systemic inflammatory response at the onset of the stroke [85,86]. In humans, a pulmonary infection during the first 15 days after stroke increases the likelihood of developing Th1 response to MBP and GFAP [87]. A more robust immune response to MBP (and to a lesser extent to GFAP) is associated with a poor outcome at this time point [88]. Such interdependence is further supported by the findings from the Planas *et al*. study indicating that increased reactivity to brain-derived compounds in cervical lymph nodes and palatine tonsil is correlated with worse outcome at follow-up [84]. Pulmonary infections predominantly caused by Gram-positive organisms are associated with the Th1 response, corresponding to a fatal course of the stroke. In contrast, urinary tract infections caused primarily by Gram-negative pathogens usually do not induce Th1 response and are associated with a better prognosis [86]. It is therefore possible that pulmonary infection, despite immunosuppression, provokes an inflammatory response strong enough to upregulate the bystander adaptive autoimmune response to brain antigens in either peripheral lymphoid organs or in the brain itself [87]. However, it cannot be excluded that the better prognostic consequences of urinary infections may also be related to their lower severity and mortality and not only to differences in their potential role of favoring autoimmunity after stroke.

## Conclusions

**Figure 2:**
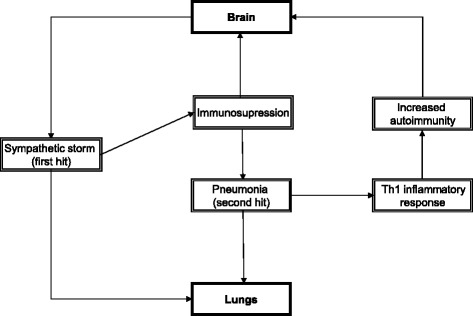
**Vicious circle of brain-lung inflammation during ischemic stroke.**

Current evidence suggests that immune-sympathetic interplay is a key point in understanding the dynamic environment of ischemic stroke. The lungs are both strongly affected and may actively participate in this interplay. We propose a model that links the ‘double hit’ and ‘Th1 response to pneumonia’ theories (Figure 2). The initial sympathetic storm directly affects the lungs (first hit), and indirectly makes them more susceptible to infection due to immunosuppression (second hit). Immunosuppresion shifts the immune response from Th1 to Th2 to protect the brain from the adaptive immune response, but pneumonia may overcome this shift, and restart Th1 bystander autoimmune response directed against CNS antigens.

In this review, we summarized the current knowledge related to the immune-sympathetic interactions with particular emphasis on the brain-lung cross-talk during this interplay. Better understanding of the pathomechanisms underlying stroke is even more urgent and important when we consider that most of the proposed experimental therapies failed to provide benefits in clinical trials in humans. In future research, data acquisition taking into account the complexity of the nervous and immune system interactions as a multisystem network most likely represents the most promising approach in describing the interrelations and pathways involved in stroke.

## Abbreviations

ALI: Acute lung injury
bFGF: basic fibroblast growth factor
β-ARs: β-adrenergic receptors
CNS: central nervous system
CCR: chemokine receptors
EGF: epidermal growth factor
GFAP: glial fibrillary acidic protein
HPA: hypothalamic-pituitary-adrenal
iNOS: inducible nitric oxide synthase
IP-10: IFN-γ-induced protein 10
LPS: lipopolysaccharide
MBP: myelin basic protein
MCP-1: monocyte chemotactic protein
MIP-2: macrophage inflammatory protein 2
NPE: neurogenic pulmonary edema
NK: natural killer
ROS: reactive oxygen species
SNS: sympathetic nervous system
T_C_ T: cytotoxic
T_H_ T: helper
Treg: regulatory T
